# Oxidative origin of sperm DNA fragmentation in the adult varicocele

**DOI:** 10.1590/S1677-5538.IBJU.2019.0827

**Published:** 2021-02-03

**Authors:** Jessica Timóteo Jeremias, Larissa Berloffa Belardin, Fatima Kazue Okada, Mariana P. Antoniassi, Renato Fraietta, Ricardo Pimenta Bertolla, Paula Intasqui

**Affiliations:** 1 Universidade Federal de São Paulo - Unifesp Divisão de Urologia Departamento de Cirurgia São Paulo SP Brasil Departamento de Cirurgia, Divisão de Urologia, Seção de Reprodução Humana, Universidade Federal de São Paulo - Unifesp, São Paulo, SP, Brasil

**Keywords:** DNA Fragmentation, Infertility, Male, Oxidative Stress

## Abstract

**Purpose:**

Sperm DNA fragmentation is a major cellular mechanism underlying varicocele-related male infertility. However, the type of DNA fragmentation - whether oxidative or of another nature - remains unknown. Thus, the aim of this study was to evaluate single- and double-stranded sperm DNA fragmentation, and oxidative-induced sperm DNA damage in men with varicocele.

**Materials and Methods:**

A cross-sectional study was performed, including 94 normozoospermic adults, of which 39 men without varicocele (controls) and 55 men with varicocele grades II or III, uni- or bilaterally. All men collected semen by masturbation. After semen analysis, the remaining volume was used for evaluation of three types of sperm DNA damage: (i) total DNA fragmentation, using an alkaline comet assay, (ii) double-stranded DNA fragmentation, using a neutral comet assay, and (iii) oxidative DNA damage, using an alkaline comet assay associated with the DNA glycosylase formamidopyrimidine enzyme. In each assay, percentage of sperm with any degree of DNA fragmentation, and with high DNA fragmentation were compared between the groups using an unpaired Student's t test or a Mann-Whitney test.

**Results:**

The varicocele group presented a higher rate of sperm with fragmented DNA (both any and high DNA fragmentation), considering single-stranded DNA fragmentation, double-stranded DNA fragmentation, or a combination of both, as well as oxidative- induced DNA fragmentation.

**Conclusions:**

Patients with varicocele have an increase in sperm DNA fragmentation levels, particularly in oxidative stress-induced sperm DNA damage.

## INTRODUCTION

Varicocele, considered the main treatable cause of male infertility, is characterized by venous dilation in the pampiniform plexus with blood reflux (1). Varicocele is associated with semen alterations (2–4), however, semen analysis itself does not provide a direct diagnosis of infertility, due to its low sensitivity (5). Therefore, additional methods to assess sperm quality have been studied and, particularly in varicocele, sperm functional abnormalities, such as increased sperm DNA fragmentation, have already been observed (2, 6).

Sperm DNA fragmentation is considered one of the main cellular mechanisms associated with male infertility. Studies have demonstrated that it is associated with decreased pregnancy rates, both in natural and assisted reproduction, with increased pregnancy loss, and with poor embryo development (7, 8). Approximately 10% of sperm from fertile men and 20-25% of sperm from infertile men present DNA damage (9). Sperm DNA fragmentation may occur mainly due to apoptosis during spermatogenesis, leading to double-stranded DNA damage (DSB), in chromatin packaging during spermiogenesis, which generates single-stranded DNA breaks (SSB), and due to oxidative stress (10).

Among the many causes of sperm DNA fragmentation in men with varicocele, oxidative stress is suggested as one of the most important factors (11). It is defined as an imbalance between the production of reactive oxygen species (ROS) and antioxidants, in favor of the oxidant (12). Indeed, an increase in sperm ROS levels, as well as a decrease in total antioxidant capacity in the seminal plasma of men with varicocele were observed, along with high sperm DNA fragmentation (13, 14). Furthermore, higher levels of malondialdehy-de-a by-product of the lipid peroxidation caused by oxidative stress - were observed both in blood and seminal plasma of men with varicocele, associated with a higher percentage of sperm with fragmented DNA (15). Thus, this suggests the involvement of oxidative stress in the sperm DNA damage observed in these men, as reviewed by Majzoub et al. (16).

Oxidative stress-induced sperm DNA damage is caused by several mechanisms, both in the testes, the sperm transit throughout the male reproductive tract, and after ejaculation (17). ROS lead to the formation of 8-OH-guanine and 8-OH-20-deoxyguanosine, which thereafter promotes SSB. Furthermore, the formed hydroxyl radicals and other lipid peroxidation by-products, such as 4-hydroxy-2-nonenal, activate caspases and endonucleases (18), which, in turn, alter nucleotides and peptides functions, indirectly inducing DSB (19). Moreover, hydroxyl radicals could also lead to loss of selective membrane permeability, allowing ROS to enter the intracellular environment and, thus, to directly attack DNA (20). Finally, ROS can compromise chromatin compaction during spermiogenesis, in which DNA is more susceptible to damage due to the replacement of DNA-associated histones by protamines (21).

It has been considered that SSB can be more easily repaired by the oocyte after fertilization than DSB (10). On the other hand, although DSB can be repaired, when the damage is too extent to be repaired, or if the oocyte fails to repair the damage, it may impair embryo development and lead to a miscarriage (10, 22–24). It has been shown that infertile men present increased DSB, compared to fertile controls, which was predictive of Intracytoplasmic Sperm Injection (ICSI) failure (25). Furthermore, it has been identified that men from couples with recurrent pregnancy loss have increased DSB (24). On the other hand, analyzing both single- and double-strand breaks is more sensitive in predicting natural pregnancy (26, 27). Therefore, given the difference in prognosis of SSB and DSB, to establish the type of sperm DNA damage in men with varicocele is clinically of great importance.

Although there is an association between varicocele and increased sperm DNA fragmentation rates, the type of the damage - whether oxidative or of other nature - remains unknown. Thus, the aim of this study was to evaluate SSB and DSB levels and oxidative-induced sperm DNA damage in men with varicocele.

## MATERIALS AND METHODS

### Patients

The present study protocol was reviewed and approved by the institutional review board from our university (Reg. No. 26931314.5.0000.5505). Informed consent was submitted by all subjects when they were enrolled, and the principles of the Helsinki Declaration were followed. All reagents were obtained from Sigma-Aldrich (Missouri, USA), unless otherwise described.

A cross-sectional study was performed including 94 volunteers who attended our Andrology Laboratory, between January and September 2015. Inclusion criteria were as follows: men aged between 18 and 50 years old, whose semen presented volume ≥1.5mL, concentration ≥15×10^6^ sperm/mL, sperm progressive motility ≥32% and sperm morphology ≥4% of normal cells. Exclusion criteria were: obesity (body mass index - BMI >30.0), smoking habits or use of psychotropic substances, report of fever within 90 days prior to semen collection, medical history of alterations associated with the urogenital tract, of systemic diseases such as cancer, or of chemotherapy or radiotherapy treatments. For the control group (n=39), only individuals without varicocele were included, whereas for the varicocele group (n=55), only men with varicocele grades II or III, uni- or bilaterally, were included. Varicocele was evaluated by scrotal palpation in a temperature-controlled room with adequate illumination, and graded according to Dubin and Amelar (28):

- Varicocele grade I - dilation of spermatic cord palpable only with Valsalva maneuver;
- Varicocele grade II - dilation of spermatic cord easily palpable, with the patient standing, demonstrating marked venous dilation during Valsalva maneuver;
- Varicocele grade III - massive dilation of spermatic cord easily visualized with patient standing and intensified ectasia during Valsalva maneuver.

### Study Design

**Figure 1:**
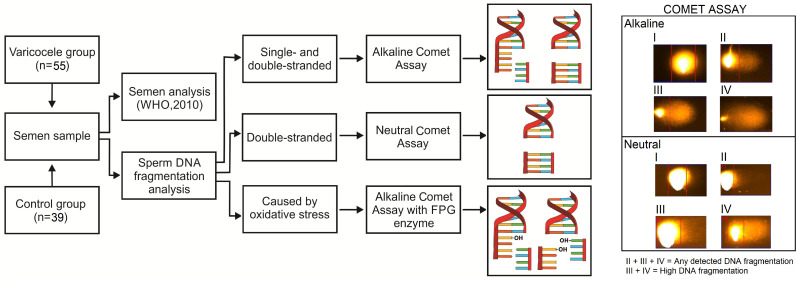
Study design, including all performed sperm DNA fragmentation tests, as well as the type of fragmentation detected in each of these tests. Right panel: representative images of cell classification, both for alkaline assays and neutral comet assay. Class I - High DNA integrity (no DNA migration); Class II - Low DNA fragmentation (an intense nucleus with little DNA migration); Class III - Increased DNA fragmentation (an observed nucleus, but with intense DNA migration); Class IV - High DNA fragmentation (an intense DNA migration and no observed nucleus). FPG - DNA glycosylase formamidopyrimidine enzyme.

Semen samples were collected at the Andrology Laboratory, by masturbation after 2 to 5 days of ejaculatory abstinence. The study design is represented in Figure-1. After semen liquefaction, an aliquot was used for semen analysis, according to the World Health Organization (WHO) recommendations, 2010 (29). The remaining semen volume was used for the following analyses: (i) total sperm DNA fragmentation (both SSB and DSB of any origin - alkaline comet assay), (ii) only DSB of any origin (neutral comet assay), and (iii) total sperm DNA fragmentation caused exclusively by oxidative stress (alkaline comet assay associated with the DNA glycosylase formamidopyrimidine [FPG] enzyme).

### Sperm DNA fragmentation analyses

To assess sperm DNA fragmentation, three different Comet assays were performed: alkaline comet assay, for evaluation of total DNA fragmentation; neutral assay to assess only DSB; and FPG-associated alkaline assay (BioLabs^®^, Ipswich, Canada). FPG is an exonuclease that recognizes and removes 8-OH-guanine and 8-OH-20-deoxyguanosine adducts, and, consequently, forms DNA fragments at these oxidative sites, allowing evaluation of total DNA fragmentation caused by oxidative stress (30). Overall protocol is described in full details in previously published articles from our group (2, 31, 32).

Briefly, for each assay, two slides were prepared with 1mL of normal melting point agarose (NMPA, Invitrogen, Burlington, USA) 1% (w:v) in Tris-Borate-EDTA (TBE) solution and kept at room temperature overnight. In each slide, 100μL of low melting point agarose (LMPA, Invitrogen, Burlington, USA) 0.75% (w:v) in TBE were added to a final sperm concentration of 1×10^6^ sperm/mL of LMPA. Slides were covered with a coverslip and kept at 4°C to solidify. After 10 minutes, coverslips were removed and 300μL of LMPA 0.75% (w:v) in TBE were added. Slides were again covered with a coverslip and kept for 10 minutes at 4°C. Subsequently, coverslip was removed, and the slides were immersed in cold lysis solution (100mM Na_2_-EDTA, 10mM Tris, 2.5M NaCl, 2% [v:v] Triton X-100, 4mM DTT, pH=11.0) at 4°C for 2 hours and then washed with milli-Q water (2×5 minutes).

In the alkaline assay, after lysis, slides were maintained in an alkaline solution (300mM NaOH; 1mM Na_2_-EDTA, pH >13.0) for 20 minutes and electrophoresis was carried out for 20 minutes at 1.5V/cm and a maximum intensity of 270mA. For the neutral assay, the same parameters were used, replacing the electrophoresis solution by TBE (pH 8.2-8.4). For the alkaline assay associated with FPG, before stabilization and electrophoresis, slides were washed with NE buffer (50mM NaCl, 10mM Tris-HCl, 10mM MgCl_2_, 1mM DTT, pH 7.9, New England BioLabs^®^, Ipswich) for 5 minutes. Thereafter, slides were incubated with the FPG enzyme (1:10.000 [v:v] dilution of a 8.000 units FPG/mL, in NE buffer) for 30 minutes at 37°C. Alkaline and neutral assay slides remained at 4°C in lysis buffer while FPG slides were being incubated with the enzyme.

Finally, all slides were washed with TBE (2×5 minutes) and fixed in ethanol 70% (v:v, 1×5 minutes) and 100% (2×5 minutes). Subsequently, slides were stained with 1mL of SYBR Green solution (SYBR Green II RNA stain gel 10.000 times in DMSO), diluted 1:10.000 (v:v) in TBE, incubated in the dark for 40 minutes and washed with TBE. Analysis was performed in an epifluorescence microscope Olympus BX-51 (Olympus, Japan), and 100 cells per sample per DNA fragmentation test were classified as: class I (high DNA integrity: no DNA migration), class II (low DNA fragmentation: an intense nucleus with little DNA migration), class III (increased DNA fragmentation: an observed nucleus, but with intense DNA migration) or class IV (high DNA fragmentation: an intense DNA migration and no observed nucleus). Representative images of this classification are included in Figure-1. For statistical analysis, we considered the sum of classes II, III and IV, meaning cells presenting with any detected DNA fragmentation, and the sum of classes III and IV as high DNA fragmentation.

### Statistical Analysis

Statistical analyses were performed using SPSS (PASW) software 18.0 for Windows (SPSS, Inc., Illinois, USA). For all analyses, the Kolmogorov-Smirnov test was utilized to verify the normality of data distribution. Normally distributed variables were compared between the control and varicocele groups by an unpaired Student's T test, and data are presented as mean, standard deviation and 95% confidence interval of the mean. On the other hand, the Mann-Whitney test was applied for non-normally distributed variables, which are described as median, interquartile range, and first and third quartile values. An alpha error of 5% was adopted for all cases. For variables presenting with a statistically significant difference, Cohen's d effect size coefficient is also presented. Effect size was considered low when below 0.25, medium when d=0.5, and high when d=0.8 (33).

## RESULTS

In total, 94 patients were included in this study, of which 39 were in the control group (without varicocele) and 55 in the varicocele group. The varicocele group was composed by: 4 patients with unilateral varicocele grade II on the left, 2 patients with unilateral varicocele grade III on the left, 1 patient with varicocele grade I on the left and grade II on the right testicle, 1 patient with varicocele grade I on the left and grade III on the right, 17 patients with varicocele grade II on the left and grade I on the right, 18 patients with bilateral varicocele grade II, 3 patients with varicocele grade III on the left and grade I on the right, 3 patients with varicocele grade III on the left and grade II on the right, and 6 patients with bilateral varicocele grade III.

Results of clinical (age) and semen variables in the control and varicocele groups are shown in Table-1. Patients with varicocele presented lower sperm progressive motility when compared to controls. The evaluation of sperm DNA fragmentation levels in men with varicocele is described in Table-2. Men with varicocele presented an increase in sperm DNA fragmentation, regardless of the type of damage (SSB and DSB of any origin or oxidative-induced SSB or DSB), both when considering any DNA fragmentation or high DNA fragmentation.

**Table 1:** Clinical and semen analysis data of the control and varicocele groups. Groups were compared using an unpaired Student's T test (α=5%), unless otherwise described.

		Control Group (n=39)	Varicocele Group (n=55)	p
**Age (years)**				
	Mean; SD	34.6; 6.96	33.6; 5.96	0.482
	95% CI	32.31-36.82	32.01-35.23	
**Sperm concentration (x10^6^/mL)**				
	Mean; SD	120.4; 51.86	118.5; 69.12	0.939
	95% CI	85.52-155.20	82.93-154.01	
**Sperm progressive motility (%)**				
	Mean; SD	51.7; 8.70	46.8; 11.28	0.026*
	95% CI	48.87-54.51	43.75-49.85	
**Sperm morphology (% normal)**				
	Median; IQ	6.0; 3.00	5.0; 4.00	0.417¥
	Q1 - Q3	5.00-8.00	4.00-8.00	

**SD** - Standard deviation; 95% **CI** - 95% confidence interval of the mean; **IQ** - Interquartile range; **Q1** - First quartile (25%); **Q3** - Third quartile (75%)

¥ Groups were compared using the Mann-Whitney test

* *p* <0.05, statistically significant

**Table 2:** Sperm DNA fragmentation analyses in the Control and Varicocele groups. Total sperm DNA fragmentation (single- and double-stranded fragmentation), double-stranded DNA fragmentation, and total oxidative DNA fragmentation levels were compared using an unpaired Student's T test, unless otherwise described (α=5%).

			Control Group (n=39)	Varicocele Group (n=55)	Effect size (Cohen's d)	*p*
**Total sperm DNA fragmentation**	**Any DNA fragmentation**	Mean; SD	64.5; 17.73	72.0; 15.29	0.453	0.031*
		95% CI	58.71 – 70.21	67.83 – 76.10		
	**High DNA fragmentation**	Median; IQ	12.0; 10,00	15.0; 11,00	0.414	**0.027**¥ *
		Q1 - Q3	7.00 – 17.00	10.00 – 21.00		
**Double-stranded DNA fragmentation**	**Any DNA fragmentation**	Mean; SD	52.9; 16.37	64.2; 13.77	0.747	0.023*
		95% CI	45.19 – 60.51	57.71 – 70.59		
	**High DNA fragmentation**	Mean; SD	4.0; 3.43	7.5; 4.58	0.865	0.010*
		95% CI	2.39 – 5.61	5.31 – 9.59		
**Total oxidative DNA fragmentation**	**Any DNA fragmentation**	Mean; SD	72.7; 17.92	79.9; 13.23	0.457	0.036*
		95% CI	66.88 – 78.50	76.37 - 83.52		
	**High DNA fragmentation**	Median; IQ	13.0; 9.00	20.0; 15.00	0.609	**0.004**¥ *
		Q1 - Q3	9.00 – 18.00	13.00 – 28.00		

**SD** - Standard deviation; **95% CI** - 95% confidence interval of the mean; **IQ** - Interquartile range; **Q1** - First quartile (25%); **Q3** - Third quartile (75%)

¥ Groups were compared using the Mann-Whitney test

* *p* <0.05, statistically

## DISCUSSION

Although the exact mechanisms through which varicocele negatively affects sperm function and semen quality are still poorly understood, sperm DNA fragmentation caused by oxidative stress is suggested as a main contributor (12). However, despite the fact that both oxidative stress and DNA fragmentation have been observed in semen of men with varicocele (13), direct oxidative DNA damage has yet to be demonstrated. Therefore, the aim of this study was to evaluate a possible oxidative origin of sperm DNA fragmentation in varicocele, in order to determine the importance of oxidative stress-induced DNA damage in these patients.

In our study, patients with varicocele presented a decrease in sperm motility compared to controls. Studies have shown this effect of varicocele (2, 4–6), although others have not observed a difference in conventional semen quality in the presence of varicocele (31). It is well demonstrated that varicocele is not homogeneous in terms of generating detectable alterations in semen quality of adult men, which in turn has led to the suggestion that more sensitive tests are necessary (34). Our study was not designed to verify the effects of varicocele on conventional semen quality, but rather to compare sperm DNA fragmentation between men with or without varicocele. However, it is important to highlight that the varicocele group did present alterations on sperm motility, in addition to the observed increased sperm DNA fragmentation.

We observed that men with varicocele presented a higher rate of sperm with SSB and DSB of any origin, when compared to the control group. Furthermore, these men also presented increased oxidative stress-related DNA fragmentation. To the best of our knowledge, our study demonstrates for the first time, using a comet assay, that in normozoospermic men with varicocele, ROS are directly responsible for damages to the DNA single and double strands. This oxidative nature of sperm DNA damage in varicocele is made more important by the fact that it occurs in the gamete. Therefore, it leads to a potential transgenerational effect which underscores the importance of treating such an effect, especially because the self-feeding nature of oxidative stress - and thus oxidative DNA damage - leads to a potentiation of the number of affected sperm in an ejaculate (35). Moreover, not all oxidative damages to DNA are detectable with current tests. For example, malondialdehyde binding to DNA, which has been shown to induce transversions to thymine and transitions to adenine in bacteria, is not detected with the current array of sperm DNA fragmentation tests (36).

In a recent systematic review and meta-analysis, Kirby et al. concluded that men with varicocele who undergo surgical repair present increased odds of achieving pregnancy and live birth when compared to those who do not (37). In a comment to that article, Hockenberry and Lipshultz added that this finding demonstrates there is essentially a mis sed opportunity in diagnosing and treating varicocele (38). Indeed, the findings of our study further support the need for a formal urological evaluation and, if needed, intervention, not only with the intent of increasing pregnancy and live birth rates, but in decreasing the potential promutagenic effects of oxidative DNA damage, such as discussed above. This is especially important because we have demonstrated that increased sperm DNA fragmentation is observed even in normozoospermic men with varicocele, and these men, currently, do not fit the criteria for a varicocelectomy. It should be noted, however, that analysis of sperm oxidative DNA fragmentation, as it was performed in our study, is non-exhaustive, which is to say that, if oxidative DNA damage was definitely demonstrated in our study by the use of a modified Comet assay, it may not be the only source of DNA fragmentation that occurs in sperm of men with varicocele.

Direct ROS attack to the DNA strands may result in SSB by the formation of 8-OH-guanine and 8-OH-20-deoxyguanosine (18). Thereafter, these oxidative-induced SSB may be converted to DSB by two mechanisms. The first mechanism is that DSB is promoted by two opposite and close oxidative SSB events, which can spontaneously convert into a DSB (39), an event that leads to an estimated 10 to 50 DSB in all human cells daily (40). The second mechanism takes place during meiosis I of spermatogenesis, when a replicative polymerase encounters an oxidative SSB in the template strand and stalls, resulting in a collapse of the replicative fork and subsequent DSB formation (41). Besides these DSB formed from oxidative-induced SSB, DSB can also be caused during apoptosis (10).

However, sperm with DNA fragmentation still have fertilizing capacity. Although this capacity may be reduced, given that sperm DNA fragmentation has been negatively correlated with the reproductive success (8), DNA fragmentation could still result in fertilization. This may lead to: (i) uncompensated damage, when the oocyte repair machinery fails to repair DNA damage, and the embryo may fail to develop or be aborted naturally, (ii) compensated damage, when the oocyte repairs the SSB and DSB by polymerases before initiation of the first cleavage division, leading to a healthy offspring, or (iii) partially compensated damage, when deletions or sequence errors may be introduced because of partial oocyte repair, and abnormal offspring may then result (42). Therefore, although not affecting fertilization, sperm DNA fragmentation may impair embryo development, and such consequences emphasize the importance of determining the type of sperm DNA fragmentation in patients with varicocele. It is of note, however, that DSB are more hazardous to the cell, and may lead to consequences such as deletions (43).

In conclusion, normozoospermic patients with varicocele present an increase in sperm DNA single- and double-stranded DNA fragmentation, as well as an increase in sperm oxidative DNA damage.
